# Family caregivers’ role in dignity: A qualitative study can we change the title to : Dignity in Serious Illness: A Qualitative Exploration of Family Caregivers’ Contributions in low middle-income country

**DOI:** 10.1017/S1478951525000100

**Published:** 2025-02-21

**Authors:** Silva Dakessian Sailian, Yakubu Salifu, Nancy Preston

**Affiliations:** 1 Hariri School of Nursing, American University of Beirut, Beirut, Lebanon; 2 International Observatory on End-of-Life Care, Division of Health Research, Faculty of Health and Medicine, Lancaster University, Lancaster, UK

**Keywords:** Dignity, palliative care, family caregivers, qualitative, Lebanon

## Abstract

**Objectives:**

Dignity is perceived as being valued and respected. Maintaining dignity throughout illness is a fundamental principle of palliative care. Dignity can be influenced through family caregiver’s communication, support, and acts of empathy or indifference among other factors. The perception of dignity and the practices adopted by family caregivers to preserve the dignity of their ill relative with serious illness in Lebanon are explored in this paper.

**Methods:**

This is a part of a larger study that explored the understanding of dignity from patients’ and family caregivers’ perspectives in a palliative care context. Data collection involved in-depth interviews with 15 family caregivers. Interviews were analyzed using reflective thematic analysis.

**Results:**

Four main themes, that explained how family caregivers understand, and uphold their relative’s dignity during illness, were developed:

(a) Familial duty expressed through presence and compassion;

(b) Holistic care and financial stability;

(c) Social connection and family roles;

(d) Compassionate services and communication.

Family caregivers maintained the dignity of their ill relatives through being there, compassionate communication, supporting the personal and medical needs of the patient, and helping them preserve their family role. Family caregiving was often underpinned by religious values and a sense of duty. Compassionate services and effective communication were essential to preserve dignity of the ill relative during hospitalizations.

**Significance of results:**

Family caregivers assume multiple roles in fostering the dignity of relatives with serious illnesses. It is crucial that family caregivers are supported by policies, healthcare systems, and community initiatives as patients cannot thrive nor sustain dignity without their support.

## Introduction

Family caregivers are essential partners in the care of relatives with palliative care needs. Often, they are heavily involved in providing the physical, social, emotional, and practical needs of the relative who becomes gradually more dependent at the end of life (Glajchen 2004; Martín et al. 2016). Dignity is perceived as a basic human right involving being esteemed, valued, and respected (Chua et al. 2022). Conserving a patient’s dignity while providing care is a fundamental concern regardless of the patient’s age, diagnosis, or place of care as loss of dignity may lead to worsening perception of selfhood, demoralization and triggering the wish to hasten death (Chochinov et al. 2002; Rodríguez-Prat et al. 2017). While dignity is an inherent human trait, it is also a relational concept influenced by the caregiver’s tenor, communication style, provision of support, and acts of empathy or indifference (Chochinov 2006 {Obispo, 2022 #9507}). Dignity may be violated when a person is vulnerable especially in situations of relational conflict, isolation or disharmony with the counterpart (Chua et al. 2022; Franco et al. 2019). Concurrently, dignity may be promoted when the ill person feels socially connected, receives good care from the family caregivers, and enjoys genuine family engagement paving the way for meaningful relationships (Andorno 2009; Chua et al. 2022; Guo and Jacelon 2014; Jacobson 2009).

In Lebanon, family caregivers are deeply engaged and take charge in the caregiving role to ensure their ill relatives receive quality care (Dumit et al. 2015). The family unit is a basic social structure in the Arab world, much of Asia, and Latin Americas, often playing a key role inthe delivery of informal care, decision-making, and communication with health care providers (Osman and Yamout 2022). Dignity preserving care, maintaining the values and worth of the patient, occupies a central ground in palliative care principles, and is an imperative standard in health care policies, and human rights’ laws (Brennan 2014). Although dignity perceptions or dignity conserving interventions are often explored from the patient’s or health care providers’ perspective (Chua et al. 2022), the evidence from the family caregivers’ views is limited (Guo and Jacelon 2014). Providing a safe psychological and physical environment, sense of belonging, and social support have been identified by caregivers as promoting patient dignity (Liang et al. 2023). Whereas, abandonment, acts of omission, physical or psychological humiliation have been associated with dignity loss (Nåden et al. 2013). Exploring family caregivers’ perceptions of dignity, and unfolding the strategies they adopt to preserve it, is vital to understand their role in enhancing the dignity of the ill relative with palliative care needs.

This study is part of a larger study that aimed to explore dignity perceptions from patients’ and family caregivers’ perspective within palliative care setting in Lebanon (Sailian Sap et al. 2022). This paper presents the perceptions of dignity and the practices that family caregivers adopt to preserve the dignity of their ill relative.

## Methods

This is a qualitative study adopting the social constructivist paradigm. A social constructivist lens assumes that perceptions of dignity and practices that enhance or demean it are the outcome of a social reality, co-constructed between the participant and the researcher within existing sociocultural norms (Burr 2015). The findings are reported in accordance with the Standards for Reporting Qualitative Research guidelines (O’Brien et al. 2014).

### Setting

The study was conducted in Beirut, Lebanon, during the COVID-19 pandemic and political uprising (Chulov 2019). The settings were 2 sites providing care to patients with chronic, advanced or terminal illnesses: a health clinic and a hospital ward of a tertiary hospital. The hospital offers in-patient palliative care services to around 400 patients per year but has no support program to the family caregivers.

### Participants

Participants were adult caregivers of relatives with serious illness related as the parent, spouse, son, daughter, or a sibling. Caregivers provided unpaid assistance with personal, psychological, household needs as well as financial support and arranging for the acquisition of health services (Roth et al. 2015). Participants needed to be actively involved in their care for at least 6 months, willing to be interviewed and capable of communicating either in English, Arabic, or Armenian. Family care givers needed to be caring for a relative at home or supporting someone in hospital. The relative with serious illness is any individual with advanced chronic or terminal illness such as cancer, organ failure, or neurodegenerative disease who has been hospitalized at least once in the past year (World Health Organization 2020). The receipt of palliative care services is not a criterion for selection.

### Sampling

Fifteen participants were sampled purposefully (Patton 2015) seeking specific variation in relationship to the relative, diagnosis, age, gender, and religion to enhance transferability. When information power, relevant to the aim of the study, was achieved, no further sampling was necessary (Malterud et al. 2016).

### Recruitment

The family caregivers were recruited from the health clinics or the hospital wards through the patients or staff. The potential participants were contacted and informed about the study details through sending the Participant Information Sheet and the informed consent document via WhatsApp, which is widely used in Lebanon, for familiarization before data collection.

Questions about the research were addressed at the beginning of the interviews. The voluntary nature of participation and the right to withdraw was discussed before oral consent was secured and recorded for all the interviews due to the inability to interview all patients in person.

### Data collection

Eight semi-structured interviews were conducted via telephone by SDS due to COVID-19 compulsory social isolation, 1 in person in a hospital setting and 6 in the participants’ homes in person. The interviews were conducted between September 2020 and April 2021 and audio recorded. They lasted a median of 30 minutes. Field notes were taken to capture initial impressions and thoughts of the researcher. The topic guide (online supplemental appendix 1), utilized during the interviews was prepared based on clinical experience and a review of the literature (Guo and Jacelon 2014). Questions included: How would you define the concept of dignity? In what ways did your relative’s illness impact their sense of dignity? How did interactions with health care providers affect your relative’s dignity? What actions do you take to support and uphold your ill relative’s dignity? What factors or practices may either decrease or enhance the dignity of your relative? Probes were used to clarify interviewees’ answers as needed.

### Reflexivity

Given the context of conducting this study in Beirut, Lebanon, during the COVID-19 pandemic and political uprising, reflexivity was crucial to acknowledge the primary researcher’s (SDS) position as a female nursing instructor of Lebanese Armenian origin who is trained in patient interviewing. Reflexivity was employed throughout the interviewing and analysis phases to mitigate this positionality and ensure a balanced interpretation of the data. Being mindful, continuous appraisal of the context, and note-taking at different phases of the research process helped in being thoughtful and harness reflexivity.

### Data analysis

The interviews were translated from Arabic to English, transcribed verbatim by SDS, so they can be uploaded to NVivo qualitative software for data management. To protect confidentiality, the interview texts were de-identified for each interview. Reflexive thematic analysis was employed to ensure a thorough and nuanced understanding of the data, involving continuous engagement and immersion by the primary researcher, SDS (Braun et al. 2019). This process included coding data line by line to identify over 200 codes, and inductively developing and refining themes that faithfully reflected the participants’ language and experiences. The data and the analysis were discussed among the researchers until the themes were refined in consensus. The open, flexible, and iterative nature of this analysis allowed for the identification of 4 main themes, ensuring the findings were substantiated with the participants’ perspectives while acknowledging the researcher’s influence (Braun 2024).

## Results

The 15 participants were from diverse religions and ethnic backgrounds (see Table 1). The majority were female (*n* = 11) reflecting the high involvement of women in caregiving. While many male caregivers supported the care financially, some of those interviewed also served as hands-on caregivers. Most participants lived in Beirut, except 2 in the suburbs.

**Table 1.** Demographics of the participants

Characteristics	Number
Caregiver relation to the relative	
Wife	2
Husband	1
Daughter	8
Son	3
Sister	1
Gender	
Male	4
Female	11
Age	
40−49	5
50−59	4
60−69	2
70−79	1
80−89	2
Type or place of interview	
Telephone	8
Home	5
Hospital	1
Reported medical condition of the patient	
Organ failure	5
Cancer	6
Neurological	4
Religion	
Christian	8
Muslim	7
Ethnicity	
Lebanese	11
Lebanese of Armenian origin	4

The participants shared their understanding of dignity and discussed their efforts in preserving the dignity in their ill relatives. Four themes on dignity were developed: (a) familial duty expressed through presence and compassion; (b) holistic needs and financial stability; (c) social connection and family roles; and (d) compassionate services and communication. These 4 themes and their subsequent subthemes described in Table 2 are interconnected as they collectively address the multifaceted nature of dignity in caregiving, encompassing emotional support, comprehensive care, social structures, accessibility of resources, and communication, all of which are crucial for maintaining the dignity of ill person. The themes are described below, supported with quotations.

**Table 2.** Themes and subthemes

Themes	Subthemes
“Familial duty expressed through presence and compassion”	Compassionate communication and attentive presence uphold dignity.
	Perseverance in care is fueled by religious values and social expectations.
	A sense of shared dignity between the patient and the spouse.
“Holistic care and financial stability”	Physical cleanliness, neat appearance, and health care support of the patient uphold dignity.
	Inability of self-care and dependence diminishes dignity.
	Dignity is enhanced by reciting the patient’s personal achievements and economic prosperity.
“Social connection and family roles”	Patient’s dignity is affirmed at home, staying connected and living within the social role of the family.
	Dignity boosted through maintaining the essence of relationships regardless of illness induced changes.
	Concealment of diagnosis and prognosis to maintain patient dignity.
“Compassionate services and communication”	“Wasta-” (intermediary network) provides easy access to health institutions and upholds dignity.
	Attentive and prompt health care services that are equitable and equal quality to all patients regardless of social rank.
	Invasive management without prior discussion of alternative solutions diminish patient dignity.
	Medical terminologies utilized by health providers increase the power gap reducing patient dignity.
	Financial capacity to pay for medical expenses enhances dignity.

### Theme 1: Familial duty expressed with presence and compassion

This theme highlights that family caregivers enhance dignity through compassionate communication and an attentive presence. Their perseverance is fueled by religious values, duty and social expectations, with time leading to a sense of shared dignity between the ill person and the spouse.

According to family caregivers, kind, compassionate communication, and staying close to the patient, especially during times of acute illness, lay the foundation for dignified care. They perceived caregiving as a familial duty and a commitment that affirms their devotion to religious beliefs, faith, and fulfills social expectations. Soothing communication, use of gentle words, and maintaining respect were regarded the basis for nurturing a dignified life for the ill relative.

So, since my aunt is now passing through this hard situation, so… we need to be by her side. “Ma feena nofrot kelna” (we can’t break down all of us), we can’t be weak or divided, she is passing through a tough period, and we have to help her. (P08)

Despite sometimes the heavy burden of caregiving, preserving the ill relative’s dignity was of utmost importance as it enhanced their reputation and social status in the community. Family caregivers spent days and nights with the ill relative during hospitalizations, bearing sacrifices to ensure presence, support, and safeguard dignity. Caregiving was a collective family responsibility and a social obligation, exhibited by the division of tasks amongst each other.

As I accepted the sweet days …., I must accept the bitter or the bad, because this can be the path to spiritual salvation from many things in our lives. So, I accept it’.…So, preserving his dignity is by not using bad language, but only kindness and sweetness is my duty towards him (P02)

Dignity was perceived as a shared concept between spouse caregivers and their partners with the state of one’s dignity affecting the other. For instance, a wife feels happy when her husband is well dressed, or washed clean because his wellness reflects on her own well-being and dignity.

But I am doing this (caregiving) for God’s sake and for my own honour. I must do this.… If someone came and saw his pyjamas and underwear dirty, his feet dirty, they would say, ‘what is this woman? She doesn’t take care of her husband’. (P04)

Family caregivers considered the dignity of their ill relative a valuable matter to attend to through practicing kind and compassionate talk, and by being next to the ill person throughout the illness.

### Theme 2: Holistic needs and financial stability

Physical cleanliness, neat appearance, health care support, reciting the patient’s personal achievements and ensuring economic prosperity uphold dignity, while inability to self-care, physical dependence, and limited purchasing power diminishes it. Family caregivers expressed that dignity is maintained through attending to the physical, psychological, emotional, and health needs of their relatives. They provided essential support like physical care, social entertainment, feeding, and securing health care supplies such as medicine and oxygen. Family caregivers aimed to ensure their ill family members are content, comfortable, clean, well fed, and symptom free at home.

One time he was begging for oxygen… so I think he felt bad, hmmm because of the illness of course, and the scarcity of resources. So yes, the illness, the pain, the shortness of breath, hm shake his dignity. (P07)

Of course, of course. My mother’s dignity is a priority to me, that she doesn’t feel upset. I make sure that all her needs are met, that she has nothing to complain about. (P09)

Losing functionality and the ability to take charge of basic personal care compromises relative’s dignity. Family caregivers offer personal assistance only after receiving the relative’s approval to reduce feelings of incompetence. For example, a daughter asks her mother’s permission before giving her a bath to preserve her dignity.

I tell her that, I just want to help and see if she wants, she accepts my help. I ask her first “can I do this?” “Let me help you with this.” “Do you want somebody to be with you?” (P14)

When she can’t reach the toilet in time, this affects her badly. (P12)

Most family caregivers expressed that recalling and celebrating the life experiences and accomplishments of the relative boost dignity and respect. Hence, they tend to recite those achievements during social encounters such as gaining high-community status, accumulated wealth, or a good reputation at work to affirm their capabilities and dignity. A male caregiver explained that he lifts his mother’s dignity through affirming and praising her cooking skills.

-
  If I am around, I pass by for a cup of coffee, you know, these things will make her always happy …. and I always try to eat something from her kitchen, and I remind her that her food is the best, this makes her smile and then everything will be fine. (P09)

Economic prosperity is also seen crucial to assure all needs of the ill relative is met and dignity preserved. Asking for monetary assistance may imply weakness and diminish dignity of both the ill relative and family caregiver potentially leading to social isolation. Poverty and the inability to secure a good quality life do not resonate with dignity as it signifies low social status and vulnerability.

Don’t ask for monetary assistance from anyone. If you ask for assistance people will run away from you and gossip … they will isolate you, and not relate to you. (P04)

Preserving the physical needs of the relative through maintaining cleanliness, securing daily basic and medical needs, ensuring functional control over personal activities and providing moral support were strategies adopted by family caregivers to enhancing dignity in the ill relative.

### Theme 3: Social connection and family roles

This theme affirms that relative’s dignity is acknowledged at home by staying socially connected and maintaining their role within the family hierarchy. Dignity is boosted through preserving relationships regardless of illness induced changes and concealing diagnosis and prognosis when necessary.

Maintaining their ill relatives’ role within the family, society, and community were seen as important. Dignity does not exist in isolation but is deeply seated within the collective societal values and connectedness of the family fostering identity and meaning through sharing personal stories, experiences, laughter, and dreams. It is within this communicative network that an individual establishes dignity.

For me, his dignity is above all dignity, so that he stays as he was before, productive, father of his children, and a giving human being, so his role is not finished in his life. (P03)

Family caregivers explained that although the relative’s character often changes with the illness, such as becoming more childish or impatient, this is overcome with humor, gentle communication, reinstating the individual’s inherent family role, safeguarding dignity.

He is still the caretaker and the head of the family; he says to us “do this … do that” he wants to be in control of us that makes him feel better. And this is true for my father and my mother. (P12)

Due to the stigma of the illness, some family caregivers did not disclose the truth about a poor diagnosis or prognosis to the relative to avoid psychological distress. Instead, they used softer words to explain the condition such as “infection” or “growth of a lump” to replace “cancer.” Some family members avoided spreading the news of the illness in the community to protect the relative’s dignity through concealment.

I won’t tell her (patient) about the metastasis… I will tell her that there is some residual ‘lump’ showing from the old thyroid … I will tell her but not everything at once…. Early morning, I was down at the pier crying … I don’t want the news to be spread. (P10)

However, family caregivers of relatives with non-cancer conditions, and those who experienced a sense of preparedness towards their death, had no problem in disclosing the medical condition.

I want them to fix her heart. They fixed it, may be temporarily, I think … I still don’t know when her heart will.. do that rhythm again. …there is always a question mark on her heart. (P06)

The ill relative’s dignity was unescapably seen as enhanced within the family setting that provided support and reinforced familial identity. Disclosing a poor prognosis was a sensitive issue to some family caregivers as it was perceived to jeopardize relative’s dignity.

### Theme 4: Compassionate services and communication

This theme illustrates features of health care services that enhance or diminish patient dignity, from the perspective of the family caregivers. Economic stability, easy access to quality health care services, ability to pay, and attentive and prompt care regardless of social rank are critical elements to maintain dignity during acute phases of illness. On the other hand, invasive medical management without prior discussion of alternative solutions, and unclear medical terminologies utilized by health providers increase the power gap reducing patient dignity.

Family caregivers found that personal contacts, “wasta-” (intermediary network), were key human acquaintances that enabled patient access to health services especially during times of limited resources. “Wasta” is a common social norm in Lebanon that consists of an informal network that facilitates in reconciling a conflict or achieving a goal. Those who were not connected to a political party or an influential leader or did not have “wasta” suffered from delayed hospital admissions or did not receive care due to limited resources.

Doctors should give equal time to everybody, not that because X person works in a famous company, they give him more time and talk to him leisurely, on the other hand they pass by quickly on a patient who is poor or from low class. (P13)

Family caregivers sought health providers who genuinely understood and addressed their relative’s needs. A health care provider who is approachable, listens, and shows readiness to provide care regardless of social rank was regarded to be fostering dignity. Practices that demeaned dignity were staff being unresponsive to call bells, unavailability of health professionals, poor coordination among health disciplines, and inconsistent care standards. Gentle humor, amiable and positive conversations were perceived to be empowering and uplifting dignity.

So, my father needed to walk. One time, two nurses came in and started joking with my father. They said let’s go have some fun, let’s have a walk around, and they took him by his hand, for a stroll and then brought him back … hmmm … and he was doing it with a smiling face. Yep. I think they really cared about him, and they were very cautious that he doesn’t get angry or upset and feel safe with them. (P08)

Family caregivers expected health care providers to explore alternative measures before resorting to artificial or invasive interventions. Applying hand restraints or inserting a nasal feeding tube without negotiating with the family, were regarded as imposing, violating patient dignity.

The doctor told me we want to insert a tube from her nose for her feeding, because she is not eating (upset), but a friend, …. told me that I can get a syringe instead and put the smashed food inside it, and then I can feed her like that. I did that and it worked. I am grateful for her. (P12)

It was unnecessary (the restraints). We could have resolved it if they had called us, and that made it worse for her. (P01)

Family caregivers also requested clear, simple explanations of the health condition without medical jargon. A prescriptive language that holds no space for dialogue and employment of multiple medications was not welcomed and sometimes was seen as criticism.

When doctors don’t say everything about the case or use language we don’t understand, as if he has more power, this is bad. (P01)

Look sometimes my father has very poor compliance in his diabetic journey, when his physician gave him various instructions and comments about his lifestyle, inferring that he (my father) is not compliant and that he should do something about it eh … he got annoyed of it, he didn’t accept, and it felt offensive to him. (P04)

Family caregivers described distress over hospital expenses, which increased the burden and affected the dignity of both the relative and themselves. Financial strain was a significant barrier to dignity.

Last time, my daughter told me I paid the bill of the hospital, but they called me again asking for another payment. I told them my daughter had ‘closed’, paid the bill and paid all our dues, they told me no, you need to pay for the exit charge and the cost of the documents we used. I didn’t have the amount, I had only …., this is my day’s money (for daily living), I told them if you accept take it or else keep the patient with you. This is the worst thing that has ever happened (angry). (P11)

Health care services that were affordable, accessible and person-centered were regarded to be necessities that enhanced dignity in the ill relative during health care encounters. Family caregivers needed to be aware of the patient condition and the plan of care before its implementation.

## Discussion

Family caregivers enhance patient dignity in multiple ways. Addressing patient’s holistic needs, preserving the social role of the relative within the family, maintaining financial stability, and following up on medical needs and consults, are all strategies adopted by family caregivers to ensure their ill relative remains dignified. As elaborated in other studies of dignity in serious illness, accessible health services, compassionate caring culture, social inclusion and fellowship, relational interactions, and clear communication were identified as essential to preserve patient dignity during acute illness (Sailian et al. 2024; Tranvåg et al. 2015).

Commitment and loyalty in caregiving means that family caregivers in Lebanon uphold patient dignity through being present and providing support with kindness and a gentle approach. Lebanon’s cultural values, rooted in tradition and religion, underscore the significance of close family ties and moral obligation shaping the caregiving process (World Values Survey 2020). Caregiving in Lebanon shares common values to countries in northern Europe like Lithuania, where it is underpinned by familial duty, to sustain the ill relative physically, emotionally, and economically during illness (Kuznecovienė et al. 2022). Also, in eastern cultures like China and Indonesia, family caregiving is a social and cultural tradition, fueled with the desire for mutual family support, sharing tasks, making sacrifices, and emotional connectedness, to invoke peace of mind and attain moral comfort and social approval (Chan et al. 2012; Kristanti et al. 2019).

The need for affirmation of the social role of the ill person in the family as a father, a mother, and ascertaining its entrenched value was often practiced by the family caregivers to preserve dignity. The concept of social connectedness and informal networks was central in reassuring, consoling and restoring the ill relative’s self-esteem, enhancing dignity comparable to other Middle Eastern countries like Iran (Babaei and Abolhasani 2020) and Asian cultures (Lou et al. 2021). Similar to the findings of the meta-synthesis (Liang et al. 2023), ill relatives are supported at home connected with family members to enhance feeling of “belonging” maintaining their role in the family and wider society, as the home environment serves as a source of dignity where their achievements, personal qualities and pride are acknowledged.

Family caregivers need accessible health care services that are equitable and affordable to all without political interference or intermediaries. They desire to have compassionate and genuinely engaged health care providers who are approachable and skilled in effective communication. Similar to European or other Middle Eastern countries, giving clear information, explaining the plan of care or future expectations in simple words, providing psychological support are deemed critical to a person’s dignity as well as for a sense of security to family caregivers (Babaei and Abolhasani 2020; Robertson et al. 2022). In contrast, as reported in Middle-Eastern studies, communication limited to merely medical details or prescriptions, without addressing the psychosocial needs of the patient, was regarded to be too technical, bewildering, and unsatisfactory (Sailian et al. 2021). Medical terminologies increased the power gap between the patient and health provider reducing patient dignity. It is worth noting that the evidence on patient dignity with palliative needs is comparable to that of ill relatives as expressed by the family caregivers.

Family caregivers in the United Kingdom, Canada, New Zealand, and Indonesia experienced challenges when combining employment and caregiving (Gardiner et al. 2022; Kristanti et al. 2019). The demands of both roles often necessitated giving up their jobs or the introduction of significant changes for accommodation. Family caregivers wanted to remain employed to guarantee an income and secure financial capacity to have access to health services needed to the well-being of the ill relative (Gardiner et al. 2022; Kristanti et al. 2019). This is similar to the Lebanese context where financial stability was crucial to safeguard patient dignity especially in times of economic crisis and shortage as the case in Lebanon. As access to quality health care services is often limited to the socially privileged, economic stability becomes essential to secure a dignified care for people with serious illness (Sailian et al. 2024).

Similar to Asian and Arab cultures, family caregivers prefer not to disclose the diagnosis or poor prognosis to their relatives to decrease psychological distress and hence enhance dignity (Alzahrani et al. 2018; Sailian et al. 2021; Ghoshal et al. 2019; Kristanti et al. 2019). Cancer is still stigmatized in Lebanon, associated with negative discourse and misperceptions of incurability (Bou Khalil 2013). In this regard, discussions about an individual’s deteriorating health are regarded as dishonorable and damaging to a patient’s dignity.

### Implications to practice/policy

Supporting family caregivers: Since family caregivers hold a crucial and considerable role in the care of the ill relative across multiple countries globally; they have to be enabled and supported by health care professionals to maintain good caregiving that in turn preserves the dignity of their ill relative. Ensuring comprehensive support to family caregivers such as helping them retain paid employment, includes the involvement of various stakeholders like governmental policies, and community or employer initiatives, that in turn is beneficial to the ill relative, the caregiver and the society in general (Gardiner et al. 2022; Kuznecovienė et al. 2022). This may be accomplished through needs assessment and support programs such as direct caregiver support, timely advice and referrals, assurance, communication with health care providers, and financial allowances to relieve caregiving burden (Aoun et al. 2015; Gardiner et al. 2022).

Clear communication from health care providers: Health care providers need to be aware and trained to employ compassionate communication using simple and clear language when conversing with family caregivers of ill relatives. Discussing interventions, particularly intensive ones, prior to their implementation is critical to ensure the ill person and the family caregivers receive care that is concordant to their wishes and perceived as dignified.

Financial stability and equal accessibility of health services: Family caregivers express the need for financial stability to follow up with the medical needs, supplies and services required for the care of their ill relatives. Having a national coverage of health services or adequate palliative care services and financial reimbursement will relieve family caregivers from some of the economic burden. Quality health services need to be accessible equally to all who are in need without discernment or discrimination. These changes would involve changes at the systems and governmental level.

### Strengths and limitations

This study provides rich insights from family caregivers’ perspectives on how dignity is enhanced in relatives with various serious illnesses, illuminating differences about dignity from the Eastern Mediterranean region. It emphasizes the key role of family caregivers in maintaining dignity both at home and during hospitalization. However, it did not address dignity from a health care provider or policy maker perspective as well. The findings provide contextual insights which highlight the needs of family caregivers, particularly that of financial security, the need for support, and clear communication from health providers, during the caregiving journey in maintaining their relative’s dignity.

The COVID-19 health crises as well as severe economic crash of Lebanon could be a limitation of this study since the study was conducted during this period. Thus, participants’ views could be influenced by the unprecedented period of socio-political uncertainty and health care shortage. Nonetheless, these findings provide an initial understanding of the role and perceptions of family caregivers in enhancing dignity in a context where collective family decision-making is valued. Further research such as longitudinal studies could unveil how the role of family caregivers changes with the intensity and trajectory of the illness and how they shape end of life of their relatives.

In conclusion, dignity is upheld by family caregivers through their support and attendance to the holistic well-being they provide to their ill relatives. Preserving dignity is perceived as a duty and a moral obligation for the family caregiver and the family, is an important social unit, and a source of comprehensive support for the person who is unwell. However, family caregivers find it challenging in maintain this culturally desirable duty when faced with challenges of financial support, access to health services, and effective communication with health providers. It is important to ensure that family caregivers are supported so they continue in safeguarding the dignity of their relatives.

## Supporting information

Sailian et al. supplementary material Sailian et al. supplementary material

## Supplementary material

The supplementary material for this article can be found at https://doi.org/10.1017/S1478951525000100.

## References

[ref1] Alzahrani AS, Alqahtani A, Alhazmi M, et al. (2018) Attitudes of cancer patients and their families toward disclosure of cancer diagnosis in Saudi Arabia: A Middle Eastern population example. *Patient Preference and Adherence* 12, 1659–1666. doi:10.2147/ppa.S176651 30214168 PMC6126501

[ref2] Andorno R (2009) Human dignity and human rights as a common ground for a global bioethics. *Journal of Medicine and Philosophy* 34(3), 223–240. doi:10.1093/jmp/jhp023 19386998

[ref3] Aoun SM, Deas K, Howting D, et al. (2015) Exploring the support needs of family caregivers of patients with brain cancer using the CSNAT: A comparative study with other cancer groups. *PLoS One* 10(12), e0145106. doi:10.1371/journal.pone.0145106 PMC4682982 26679505

[ref4] Babaei S and Abolhasani S (2020) Family’s supportive behaviors in the care of the patient admitted to the cardiac care unit: A qualitative study. *Journal of Caring Sciences* 9(2), 80–86. doi:10.34172/jcs.2020.012 32626669 PMC7322406

[ref5] Bou Khalil R (2013) Attitudes, beliefs and perceptions regarding truth disclosure of cancer-related information in the Middle East: A review. *Palliative and Supportive Care* 11(1), 69–78. doi:10.1017/s1478951512000107 23171758

[ref6] Braun V, Clarke V, Hayfield N, et al. (2019) Thematic Analysis. In Liamputtong P ((ed)), *Handbook of Research Methods in Health Social Sciences*. Singapore: Springer Singapore, 843–860.

[ref7] Braun VCV (2024) Supporting best practice in reflexive thematic analysis reporting in palliative medicine: A review of published research and introduction to the reflexive thematic analysis reporting guidelines (RTARG). *Palliative Medicine* 38(6), 608–616. doi:10.1177/02692163241234800 38469804 PMC11157981

[ref8] Brennan F (2014) Dignity: A unifying concept for palliative care and human rights. *Progress in Palliative Care* 22, 88–96. doi:10.1179/1743291X13Y.0000000064

[ref9] Burr V (2015) *Social Constructionism*, Third edition. London: Routledge.

[ref10] Chan S, Ho A, Leung P, et al. (2012) The blessings and the curses of filial piety on dignity at the end of life: Lived experience of Hong Kong Chinese adult children caregivers. *Journal of Ethnic & Cultural Diversity in Social Work* 21, 277–296. doi:10.1080/15313204.2012.729177

[ref11] Chochinov (2006) Dying, dignity, and new horizons in palliative end-of-life care. *CA: A Cancer Journal for Clinicians* 56, 84–103. doi:10.3322/canjclin.56.2.84 16514136

[ref12] Chochinov H, Hack T, Hassard T, et al. (2002) Dignity in the terminally ill: A cross-sectional, cohort study. *The Lancet* 360(9350), 2026–2030. doi:10.1016/S0140-6736(02)12022-8 12504398

[ref13] Chua KZY, Quah ELY, Lim YX, et al. (2022) A systematic scoping review on patients’ perceptions of dignity. *BMC Palliative Care* 21(1), 118. doi:10.1186/s12904-022-01004-4 PMC9251939 35787278

[ref14] Chulov M (2019) Lebanon’s mass revolt against corruption and poverty continues. https://www.theguardian.com/world/2019/oct/20/lebanons-mass-revolt-against-corruption-and-poverty-continues (accessed 3 August 2023).

[ref15] Dumit NY, Abboud S, Massouh A, et al. (2015) Role of the Lebanese family caregivers in cardiac self-care: A collective approach. *Journal of Clinical Nursing* 24(21-22), 3318–3326. doi:10.1111/jocn.12949 26249817 PMC4750903

[ref16] Franco SMG, Donato SCT, Carvalho RT, et al. (2019) Perception of dignity of patients in palliative care. *Text & Content Nursing* 28, e20180142. doi:10.1590/1980-265x-tce-2018-0142

[ref17] Gardiner C, Taylor B, Goodwin H, et al. (2022) Employment and family caregiving in palliative care: An international qualitative study. *Palliative Medicine* 36(6), 986–993. doi:10.1177/02692163221089134 35848213 PMC9344494

[ref18] Ghoshal A, Salins N, Damani A, et al. (2019) To tell or not to tell: Exploring the preferences and attitudes of patients and family caregivers on disclosure of a cancer-related diagnosis and prognosis. *Journal of Global Oncology* 5, 1–12. doi:10.1200/JGO.19.00132 PMC6882506 31770048

[ref19] Glajchen M (2004) The emerging role and needs of family caregivers in cancer care. *Journal of Supportive Oncology* 2(2), 145–155. PMID: 15328817. 15328817

[ref20] Guo Q and Jacelon C (2014) An integrative review of dignity in end-of-life care. *Palliative Medicine* 28(7), 931–940. doi:10.1177/0269216314528399 24685648

[ref21] Jacobson N (2009) Dignity violation in health care. *Qualitative Health Research* 19(11), 1536–1547. doi:10.1177/1049732309349809 19797155

[ref22] Kristanti MS, Effendy C, Utarini A, et al. (2019) The experience of family caregivers of patients with cancer in an Asian country: A grounded theory approach. *Palliative Medicine* 33(6), 676–684. doi:10.1177/0269216319833260 30916614 PMC6537031

[ref23] Kuznecovienė J, Butkevičienė R, Harrison WD, et al. (2022) What does it mean to be the main caregiver to a terminally ill family member in Lithuania?: A qualitative study. *PLoS One* 17(5), e0265165. doi:10.1371/journal.pone.0265165 PMC9098011 35551302

[ref24] Liang M, Xie X, Pan Y, et al. (2023) A qualitative meta-synthesis of patient dignity from the perspective of caregivers. *BMC Geriatrics* 23(1), 351. doi:10.1186/s12877-023-04071-1 PMC10243055 37277725

[ref25] Lou C, Lou K and Ridley J (2021) Exploring the meaning of dignity at end of life for Chinese Canadians caregivers: A qualitative cross-cultural study. *Palliative Medicine* 35(1), 142–150. doi:10.1177/0269216320956809 32998628

[ref26] Malterud K, Siersma VD and Guassora AD (2016) Sample size in qualitative interview studies: Guided by information power. *Qualitative Health Research* 26(13), 1753–1760. doi:10.1177/1049732315617444 26613970

[ref27] Martín JM, Olano-Lizarraga M and Saracíbar-Razquin M (2016) The experience of family caregivers caring for a terminal patient at home: A research review. *International Journal of Nursing Studies* 64, 1–12. doi:10.1016/j.ijnurstu.2016.09.010 27657662

[ref28] Nåden D, Rehnsfeldt A, Råholm M-B, et al. (2013) Aspects of indignity in nursing home residences as experienced by family caregivers. *Nursing Ethics* 20(7), 748–761. doi:10.1177/0969733012475253 23462504

[ref29] O’Brien BC, Harris IB, Beckman TJ, et al. (2014) Standards for reporting qualitative research: A synthesis of recommendations. *Academic Medicine* 89(9), 1245–1251. doi:10.1097/acm.0000000000000388 24979285

[ref30] Osman H and Yamout R (2022) Palliative Care in the Arab World. In Al-Shamsi HO, Abu-Gheida IH, Iqbal F and Al-Awadhi A ((eds)), *Cancer in the Arab World*. Singapore: Springer Singapore, 381–393.

[ref31] Patton M (2015) *Qualitative Research and Evaluation Methods.*, 4th edn. Thousand Oaks: Sage Publications.

[ref32] Robertson SB, Hjörleifsdóttir E and Sigurðardóttir Þ (2022) Family caregivers’ experiences of end-of-life care in the acute hospital setting. A qualitative study. *Scandinavian Journal of Caring Sciences.* 36(3), 686–698. doi:10.1111/scs.13025 34382701 PMC9545473

[ref33] Rodríguez-Prat A, Balaguer A, Booth A, et al. (2017) Understanding patients’ experiences of the wish to hasten death: An updated and expanded systematic review and meta-ethnography. *BMJ Open* 7(9), e016659. doi:10.1136/bmjopen-2017-016659 PMC5640102 28965095

[ref34] Roth DL, Fredman L and Haley WE (2015) Informal caregiving and its impact on health: A reappraisal from population-based studies. *Gerontologist* 55(2), 309–319. doi:10.1093/geront/gnu177 26035608 PMC6584119

[ref35] Sailian D, Salifu Y, Saad R, et al. (2021) Dignity of patients with palliative needs in the Middle East: An integrative review. *BMC Palliative Care* 20(1), 112. doi:10.1186/s12904-021-00791-6 PMC8285813 34271909

[ref36] Sailian SD, Salifu Y and Preston N (2024) Dignity enhanced through faith & family support in palliative care: A qualitative study. *BMC Palliative Care* 23(1), 142. doi:10.1186/s12904-024-01478-4 PMC11157805 38849809

[ref37] Sailian Sap D, Preston N and Salifu Y (2022) The Dignity of Patients with Palliative Needs: Patients’ and Family Caregivers’ Perspective in Lebanon. PhD Thesis, Faculty of Health and Medicine > Health Research, Lancaster.

[ref38] Tranvåg O, Petersen KA and Nåden D (2015) Relational interactions preserving dignity experience: Perceptions of persons living with dementia. *Nursing Ethics* 22(5), 577–593. doi:10.1177/0969733014549882 25319119

[ref39] World Health Organization. W (2020) Palliative Care Fact Sheet. http://www.who.int/mediacentre/factsheets/fs402/en/ (accessed 20 December 2023).

[ref40] World Values Survey (2020) *World Values Survey, Findings and Insights*. Madrid Spain: In.: World Values Survey.

