# Dignity in Serious Illness: A Qualitative Exploration of Family Caregivers’ Contributions in low middle-income country – ERRATUM

**DOI:** 10.1017/S1478951525000288

**Published:** 2025-03-13

**Authors:** Silva Dakessian Sailian, Yakubu Salifu, Nancy Preston

**Keywords:** Dignity, palliative care, family caregivers, qualitative, Lebanon, erratum

https://doi.org/10.1017/S1478951525000100, Published by Cambridge University Press, 21 February 2025.

The Publisher apologizes for publishing this article with an incorrect article title. The title should have been:

Dignity in Serious Illness: A Qualitative Exploration of Family Caregivers’ Contributions in low middle-income country

This has now been corrected in the published article.
