# Supplementary material for: Family caregivers’ role in dignity: A qualitative study can we change the title to : Dignity in Serious Illness: A Qualitative Exploration of Family Caregivers’ Contributions in low middle-income country
Source: Palliat Support Care. 2025 Feb 21;23:e61. doi: 10.1017/S1478951525000100 (PMC13166562; doi:10.1017/S1478951525000100)
Supplement: Sailian et al. supplementary material [file S1478951525000100sup001.docx]

# **Appendix 1**

# Topic Guide

| Interview stage | Questions |
| --- | --- |
| Introduction | My name is Silva Dakessian Sailian and I am conducting research project as part of my PhD dissertation. My research topic is about the understanding the meaning of dignity in patients with illness and their family members as well as factors influencing it. |
| Consent to interviewing |  |
| Consent to recording |  |
| Background and contextual | \| Tell me about yourself, your age, work, family role… Would you mind me asking about   1. How long have you been taking care of your relative, to what extent? 2. How was your experience of caregiving till now? \| \| --- \| |
| Definitional questions  The researcher will use the term used by the patient to further explore the topic | Dignity is often mentioned to be important element in quality health care for patients with advanced chronic or terminal illnesses   1. What does the term or the concept of dignity mean to you? What other terms or words would you use to describe it? 2. How would you define personal dignity or understand it? 3. Is it associated with places or people or stages in your life? Does it recollect images . . . or other words? 4. Can you tell me about what are the qualities of having or preserving personal dignity? Or what meaning does it hold to you?   I’d like to move on to your experience of relative’s dignity in illness . . . and the encounter with health care provides |
| Core Discussion using prompt and follow-up questions | 1. How did the phenomenon of dignity change if any, with your family member’s illness experience? If so how did it change, what was it related to? 2. Can you think of a time when your family member experienced loss or almost loss of dignity in his/ her illness experience (diagnosis, progression….)? How did you feel? 3. What about the encounters with the health care providers (in or outside hospital setting- with nurses, physicians etc.) did the interaction affect the dignity of your family member? Did hospital admission or hospital context affect it? If yes, how? 4. Whether there were any situations while in the hospital ward when your family member lost, or could have lost, the sense of “dignity” or word used by family member. If yes, explore: 5. What actions or experiences can you recall with nurses, physicians that challenged the dignity of your patient, or made him feel uncomfortable? 6. What actions or experiences can you recall with nurses, physicians or other health providers that made your family member feel dignified? 7. How did hospital staff affect the situation (did they make the situation better or worse), anything else that you would have liked staff to do in this situation. 8. How did losing the dignity of your sick family member or conserving it affect his/her wellbeing? 9. Some studies say that dignity is related to the physical symptoms, presence of social support and personal characteristics of the individual, do you agree? To what aspect, please elaborate 10. How do you think an individual’s gender, marital status, education, employment, financial status, affect dignity, in what capacity? |
| Closing the interview | 1. What actions or advice would you recommend to health care providers, friends or family members that could conserve the dignity of an ill person? How do you preserve the dignity of your relative? 2. Is there anything else that I didn’t ask, and you want to inform me about? Any burning issues that you would like to share with me? 3. Thank the participant and ensure he/she is comfortable and well, |
